# Association of Polymorphisms of IL-6 Pathway Genes (IL6, IL6R and IL6ST) with COVID-19 Severity in an Amazonian Population

**DOI:** 10.3390/v15051197

**Published:** 2023-05-19

**Authors:** Fabíola Brasil Barbosa Rodrigues, Rosilene da Silva, Erika Ferreira dos Santos, Mioni Thieli Figueiredo Magalhães de Brito, Andréa Luciana Soares da Silva, Mauro de Meira Leite, Flávia Póvoa da Costa, Maria de Nazaré do Socorro de Almeida Viana, Kevin Matheus Lima de Sarges, Marcos Henrique Damasceno Cantanhede, Adriana de Oliveira Lameira Veríssimo, Mayara da Silva Carvalho, Daniele Freitas Henriques, Carla Pinheiro da Silva, Igor Brasil Costa, Juliana Abreu Lima Nunes, Iran Barros Costa, Giselle Maria Rachid Viana, Maria Alice Freitas Queiroz, Sandra Souza Lima, Jeferson da Costa Lopes, Maria Karoliny da Silva Torres, Izaura Maria Vieira Cayres Vallinoto, Carlos David Araújo Bichara, Antonio Carlos Rosário Vallinoto, Eduardo José Melo dos Santos

**Affiliations:** 1 Laboratório de Genética de Doenças Complexas, Instituto de Ciências Biológicas, Universidade Federal do Pará, Belém 58255-000, Brazil; 2 Programa de Pós-Graduação em Biologia de Agentes Infecciosos e Parasitários, Universidade Federal do Pará, Belém 58255-000, Brazil; 3 Hospital Adventista de Belém, Belém 66093-681, Brazil; 4 Seção de Arbovirologia e Febres Hemorrágicas, Instituto Evandro Chagas, Secretaria de Vigilância em Saúde, Ministério da Saúde do Brasil, Ananindeua 67000-000, Brazil; 5 Laboratório de Imunologia, Seção de Virologia, Instituto Evandro Chagas, Secretaria de Vigilância em Saúde, Ministério da Saúde do Brasil, Ananindeua 67000-000, Brazil; 6 Laboratório de Pesquisas Básicas em Malária, Seção de Parasitologia, Instituto Evandro Chagas, secretaria de Vigilância em Saúde, Ministério da Saúde do Brasil, Ananindeua 67000-000, Brazil; 7 Laboratório de Virologia, Instituto de Ciências Biológicas, Universidade Federal do Pará, Belém 58255-000, Brazil

**Keywords:** IL6, IL6R, IL6ST, COVID-19, SARS-CoV-2

## Abstract

Interleukin-6 has been recognized as a major role player in COVID-19 severity, being an important regulator of the cytokine storm. Hence, the evaluation of the influence of polymorphisms in key genes of the IL-6 pathway, namely IL6, IL6R, and IL6ST, may provide valuable prognostic/predictive markers for COVID-19. The present cross-sectional study genotyped three SNPs (rs1800795, rs2228145, and rs7730934) at IL6. IL6R and IL6ST genes, respectively, in 227 COVID-19 patients (132 hospitalized and 95 non-hospitalized). Genotype frequencies were compared between these groups. As a control group, published data on gene and genotype frequencies were gathered from published studies before the pandemic started. Our major results point to an association of the IL6 C allele with COVID-19 severity. Moreover, IL-6 plasmatic levels were higher among IL6 CC genotype carriers. Additionally, the frequency of symptoms was higher at IL6 CC and IL6R CC genotypes. In conclusion, the data suggest an important role of IL6 C allele and IL6R CC genotype on COVID-19 severity, in agreement with indirect evidence from the literature about the association of these genotypes with mortality rates, pneumonia, and heightening of protein plasmatic levels pro-inflammatory driven effects.

## 1. Introduction

The most striking characteristic of severe COVID-19 is the cytokine storm, where systemic inflammatory pathways are strongly activated. Along with TNF-alpha and IFN-gamma, high levels of IL-6 became one of the most important prognostic markers in COVID-19 [1].

IL-6 is a multifunctional cytokine with both pro- and anti-inflammatory properties. An IL-6 receptor bound to the membrane of hepatocytes and some leucocyte subpopulations constitutes the classic pathway, activating intracellular signaling pathways, such as JAK/STAT, and being the gp130, encoded by IL6ST gene, responsible by the signal transduction. A second pathway, called trans-signaling, is constituted by the binding of IL-6 with a soluble receptor (sIL-6R), whose complex binds to soluble gp130 forms. The interplay between the pro-inflammatory properties of the trans-signaling pathway and the anti-inflammatory signaling is highly regulated in all inflammatory processes [2] and seems to occupy a seminal position in COVID-19.

Additional evidence of the relevance of IL-6 and their receptors in COVID-19 is the reporting of successful therapeutical use of monoclonal antibodies anti-IL-6 receptors in COVID-19, whose use has been proven to be effective in a number of autoimmune diseases [3,4].

In this context, polymorphisms at IL6, IL6R, and IL6ST genes become interesting candidates to be prognostic and predictive markers of COVID-19 severity. IL-6 pathways have been reported to modulate the expression of IL6 and IL6R genes, respectively [5], and to be associated with a number of inflammatory, autoimmune, and infectious diseases [6,7,8]. Moreover, IL6ST gene mutations can cause Hyper-IgE recurrent infection syndrome-4B, a rare recessive immunologic disorder [9], highlighting IL6ST as a promising candidate gene for COVID-19.

Hence, the present study aims to evaluate the role of polymorphisms at IL6, IL6R, and IL6ST in COVID-19 severity aspects, along with their influence on IL-6 plasma levels during active COVID-19.

## 2. Materials and Methods

### 2.1. Study Design and Ethic Aspects

This is a cross-sectional study approved by the ethics committee for research involving human beings (Protocol No. 2.190.330). The sample was constituted of 227 patients collected during the period from September 2020 to July 2021. Demographic and clinical data were collected from patients, including gender, age, and COVID-19-specific data (date of onset of symptoms, symptoms at diagnosis of COVID-19, hospitalization, need for oxygen, and comorbidities). Patients were grouped according to hospitalization (hospitalized and non-hospitalized).

The same clinical parameters and health multi-professional approaches were used to evaluate and classify all patients. Both sample groups were selected from a larger sample according to very rigorous inclusion/exclusion criteria, as described below.

All patients had their diagnosis for COVID-19 confirmed by RT-PCR or antigen test. The sample included patients that are residents in Belém (Pará, Brazil) from both sexes, over 18 years old, and unvaccinated at the time of the study. The severity of acute COVID-19 was evaluated according to WHO criteria [10] from information on medical records.

The present study was approved by the National Ethic Committee (CAEE: 33470020.1001.0018; protocol number n° 2.190.330). All participants provided written informed consent. This study was conducted in strict accordance with the principles of the Declaration of Helsinki and followed recommendations provided by the guidelines for reporting observational studies, the STrengthening the REporting of Genetic Association studies (STREGA) [11].

During the first wave, which was until November 2020, we were able to collect 118 patients; 28 (24%) hospitalized. During the second wave, related to VOCs, such as Delta and Gamma (P1 lineage), that began approximately in December 2020, we sampled 105 patients. However, due to the improvement of the logistic structure, better access to hospitals, and quite a larger number of patients in hospitalized situations (it is important to highlight that while in 2020 the highest daily number of deaths reached around 1000, in the first semester 2021 reached 4000) all patients we collected were from hospitals, thus belonging to the hospitalized group.

### 2.2. Sample Processing and Genotyping

DNA was isolated from venous blood samples (4 mL) collected using EDTA as an anticoagulant. DNA isolation was performed using the kit ReliaPrepTM Blood gDNA Miniprep System (Promega), following the protocol recommended by the fabricant.

The SNPs were chosen based on their functional characteristics, most of them related to modulation of gene expression, localized in cytokine loci *IL6* (-174G/C; rs1800795); *IL6R* (358A/C; rs2228145) and *IL6ST* (55970684G>A; rs7730934). The genotyping was performed by real-time PCR using pre-designed assays (Thermo Fisher, Carlsbad, CA, EUA) in a sequence detector StepOne PLUS (Applied Biosystems, Foster City, CA, EUA), following the fabricant protocols. The assays ID are: *IL6* (C___1839697_20); *IL6R* (C__16170664_10) and *IL6ST* (C___3248953_10).

Of our 227 patients, 95 were used in a previously published study from our group [12] that evaluated cytokine levels. Thus, data on plasma cytokine levels, in particular IL-6, are available for these 95 patients that belong to the hospitalized subgroup and were collected during the active SARS-CoV-2 infection. Hence, we could test if high levels of IL-6 are associated with IL6 (rs1800795) genotypes.

### 2.3. Statistical Analyses

All SNPs were tested for Hardy–Weinberg equilibrium. The genotype and allele frequencies of each SNP were estimated by direct count. A comparison of the allele and genotype frequencies between hospitalized and non-hospitalized groups was carried out using the Fisher exact test.

In 2019, before the pandemic, a study was published investigating the polymorphism of the rs1800795 in the promoter region of the IL6 gene and its role in heart diseases associated with Chlamydia infection [13]. This study was conducted in Belém, the same population we studied, and used as control 300 healthy individuals. Thus, we used the genotypic and allelic frequencies provided by this study as representative of our population. This group was called Control.

IL-6 levels between different genotypes were compared by the Mann–Whitney test.

The frequency of symptoms between hospitalized and non-hospitalized was compared by the Wilcoxon paired test. Additionally, the frequency of symptoms was also compared between genotypes of all three SNPs.

Categorical variables, such as allele and genotype frequencies, were compared using the Fisher exact test.

Correction for multiple tests was applied if necessary and is always presented along with raw test results without correction.

## 3. Results

Clinical and demographic characteristics of the sample are presented in Table 1, while Table 2 presents the genotype and allele frequencies in the sample and subsamples.

Additionally, the allele frequencies in hospitalized and non-hospitalized groups, according to their ethnicity, are presented in Supplementary Table S1.

Table 3 presents the frequencies of COVID-19 symptoms according to SNP genotypes.

Comparison performed between genotypes revealed that carriers of the genotype IL6R CC have a higher frequency of symptoms than AA carriers (Wilcoxon paired test; Z = 2.6; *p* = 0.0092; *pc* = 0.0276); IL6 CC carriers have a higher frequency of symptoms than AA carriers (Z = 2.72; *p* = 0.0056; *pc* = 0.0168). No differences were observed between IL6ST genotypes (*p* = 0.084).

The retrieving of data on IL-6 plasmatic levels from our previous published study [12] showed that among 95 patients with active COVID-19, the levels of plasmatic IL-6 were higher among IL6 CC genotype carriers (7 patients; average 40.11 pg/mL) than among GG carriers (54 patients; average 31.2 pg/mL), making this difference statistically significant (Mann–Whitney test; Z(U) = −1.98; *p* = 0.04).

The comparison of both waves did not allow any conclusions. We compared the 28 hospitalized from the first wave with the 105 from the second wave in terms of their allelic frequencies, and no difference was found. The MAF of IL6 SNP was 21% and 28% among first and second waves patients, respectively. For IL6R and IL6ST, the differences in MAFs were even smaller. No statistical differences were detected. Finally, the symptoms profiles of hospitalized and non-hospitalized seem also similar. Hence, we opted not to address the question of differences between waves because our sampling design was not adequate for this purpose, and the original lineage and VOCs of the second wave are not so different if compared with omicron, which was detected by the end of 2021.

## 4. Discussion

In general, our data agrees, as shown by our statistical analysis, with trends from the literature in relation to higher proportions of elderly patients and comorbidity carriers in the hospitalized subsample [14,15]. In relation to symptoms, taste/smell (anosmia/ageusia) was more frequently observed in non-hospitalized patients, while respiratory symptoms (dyspnea and cough) were more frequent among hospitalized patients, corroborating the literature reports of COVID-19 [16].

The association of the allele C of the rs1800795, localized at the IL6 gene, agrees with previous meta-analyses published by some authors of our group [17]. In this study, the authors detected a positive correlation between COVID-19 mortality rates and the allele C frequency of rs1800795, suggesting that this allele could be involved in COVID-19 severity. As an exploratory meta-analysis, the results provided clues for candidate genes and SNPs, such as the eQTL rs1800795, which was confirmed by our present case-control study.

Interestingly, a significant difference was observed in Caucasian proportions, higher among hospitalized (44.4%) than among non-hospitalized (27.7%). This result might be interpreted in two ways. The first suggests that European ancestry is a risk factor for hospitalization and COVID-19 severity. The second putative implication is that the lower frequency of the G allele among those hospitalized could be a consequence of the fact that the G allele is less frequent among Europeans than among non-Europeans [18]. Indeed, the frequency of Allele G is, for example, 98% among Africans and 42% among Europeans. However, if we estimate the frequency of the G allele exclusively among the Caucasians of the hospitalized and non-hospitalized subgroups, the frequencies, 28% and 25%, respectively, are very similar, showing no statistical significance (as shown in Supplementary Table S1 [18,19,20]). The same could be observed among the other ancestry-related phenotypes (Blacks and Browns). These results suggest that European ancestry is a risk factor independent from IL6 polymorphism and that the association of hospitalization risk with allele C of rs1800795 is not a bias introduced by the higher proportion of European ethnicity among hospitalized patients.

The association of the IL6 genotype CC is also corroborated by the higher frequencies of COVID-19 symptoms among CC carriers (Table 3), as well as by the slight increase of IL-6 levels in CC in comparison to GG genotypes during the active disease.

The understanding of the functional basis of the association between the IL6 C allele with COVID-19 severity is still not clear. The role of this variant in IL6 expression is not conclusive. There is a trend toward higher IL-6 production among the allele C carriers as well as an association of this allele with more severe forms of pneumonia in general, as previously suggested in a recent meta-analysis [21]. In this context, an additional association of the IL6R genotype CC with higher frequencies of symptoms could also be observed in our results.

While the association of IL6 genotypes with serum IL-6 levels is still not completely understood, the IL6R genotype CC is more consistently associated with higher plasmatic levels of this receptor [5,22]. In particular, the plasmatic IL-6R is related to the trans-activating pathway, likely associated with pro-inflammatory response and chronic inflammatory diseases [23]. This pro-inflammatory trend is also corroborated by the successful use of IL-6R blockade by monoclonal antibodies in some inflammatory diseases [24]. Hence, some studies claim that the blockade of IL-6R could be beneficial in COVID-19 therapy protocols and even consider the influence of IL6R [3] polymorphism in these protocols [25].

Thus, the higher frequency of COVID-19 symptoms among IL6R CC genotypes could reflect the higher expression profile of this genotype and its consequent pro-inflammatory effects.

In conclusion, our results point to a consistent association of IL6 and IL6R polymorphisms with COVID-19 severity, putatively due to higher expression of these genes related to CC genotypes and their pro-inflammatory consequences, corroborating previous meta-analysis studies correlating them with mortality rates worldwide and their role in pneumonia and immunobiological therapeutic protocols involving IL-6 pathways.

## Figures and Tables

**Table 1:** Demographic, epidemiological, and clinical characterization of the sample.

Variables	Not Hospitalized n = 95 (41.85%)	Hospitalized n = 132 (58.15%)	*p*-Value (Corrected *p*-Value)
**Sex ^a^**			
Women	49 (51.58)	58 (43.94)	*p* = 0.2823 (not apply)
**Age (years) ^b^**			
18–39	50 (52.63)	29 (21.97)	*p* < 0.0001(not apply)
40–59	42 (44.21)	67 (50.76)	
≥60	3 (3.16)	36 (27.27)	
**Average**	39	51	
**Ethnicity ^c^**			
Asian	4 (4.2)	2 (1.6)	*p* = 0.2396 (not apply)
White	26 (27.7)	56 (44.4)	*p* = 0.0116 (*pc* = 0.0464)
Black	12 (12.8)	10 (7.9)	*p* = 0.2564 (not apply)
Brown	52 (55.3)	58 (46)	*p* = 0.1385 (not apply)
Not informed	1	6	
**Comorbidities ^d^**			
Yes	15 (14.79)	54 (40.91)	*p* < 0.0001 (not apply)
**Ventilatory Support**			
No	95 (100)	60 (45.45)	(not apply)
Non-invasive	0 (0.0)	70 (53.03)	(not apply)
Invasive	0 (0.0)	2 (1.51)	(not apply)
**Symptoms ^e^**			
Fever	58 (61.05)	99 (75)	*p* = 0.0292 (*pc* = 0.3796)
Cough	46 (48.42)	108 (81.81)	*p* < 0.0001 (not apply)
Coryza	38 (40)	47 (35.61)	*p* = 0.5784 (not apply)
Headache	58 (61.05)	71 (53.79)	*p* = 0.2815 (not apply)
Sore throat	40 (42.10)	43 (32.57)	*p* = 0.1663 (not apply)
Chest pain	30 (31.58)	56 (42.42)	*p* = 0.1269 (not apply)
Abdominal pain	16 (16.84)	31 (23.48)	*p* = 0.2483 (not apply)
Myalgia	51 (53.68)	79 (59.85)	*p* = 0.4147 (not apply)
Nausea and/or vomiting	17 (17.89)	45 (34.09)	*p* = 0.0069 (*pc* = 0.0897)
Diarrhea	33 (34.74)	63 (47.73)	*p* = 0.0573 (not apply)
Dyspnea	28 (29.47)	80 (60.61)	*p* < 0.0001 (not apply)
Weakness or fatigue	19 (20)	36 (27.27)	*p* = 0.2716 (not apply)
Anosmia and/or ageusia	60 (63.16)	52 (39.39)	*p* = 0.0004 (*pc* = 0.0052)

n = number of patients. ^a^ Fisher exact test; ^b^ Mann–Whitney test, Z(U) 6,5; ^c^ Fisher exact test; ^d^ Fisher exact test; ^e^ Fisher exact test.

**Table 2:** Genotype and allele frequencies of rs1800795, rs2228145, and rs7730934, localized at genes *IL6*, *IL6R*, and *IL6ST*, respectively.

Locus	Genotypes and Alleles	Frequencies (%)			
		Control (Bel)	CoV	NHSP CoV	HSP CoV
***IL6***	GG	**n (300)**	**n (227)**	**n (95)**	**n (132)**
		207 (69%) ^a^	127 (55.9%)	56 (58.9%)	71 (53.8%)
	GC	85 (28%)	86 (37.9%)	35 (36.9%)	51 (38.6%)
	CC	8 (3%)	14 (6.2%)	4 (4.2%)	10 (7.6%)
	G	83% ^a,b^	75% ^a^	77%	73% ^b^
***IL6R***	AA		**n (227)**	**n (95)**	**n (132)**
			84 (37%)	37 (39%)	47 (35.6%)
	CA		99 (43.6%)	39 (41%)	60 (45.5%)
	CC		44 (19.4%)	19 (20%)	25 (18.9%)
	C		41%	40%	42%
***IL6ST***	GG		**n (227)**	**n (95%)**	**n (132)**
			154 (67.8%)	60 (63.2%)	94 (71.2%)
	AG		61 (26.9%)	32 (33.7%)	29 (22%)
	AA		12 (5.3%)	3 (3.1%)	9 (6.8%)
	A		18.7%	20%	18%

n = number of individuals; CoV = total COVID-19 sample; NHSP = non-hospitalized patients; HSP = hospitalized patients; Control = sample representative of the city of Belém, retrieved from a study previous to the COVID-19 pandemics [13]; Significant Fisher exact test for allele frequencies comparison: ^a^ Control vs. CoV (*p* = 0.0012; *pc* = 0.0048); ^b^ Control vs. HSP (*p* = 0.0009; *pc* = 0.0036). All remaining comparisons were not statistically significant (Control vs. NHSP; NSP vs. NHSP).

**Table 3:** Frequency of major COVID-19 symptoms according to *IL6*, *IL6R*, and *IL6ST* genotypes.

Locus	*IL6R*		*IL6*		*IL6ST*	
Genotype	AA	CC	GG	CC	AA + AG	GG
**N**	84	44	127	14	72	151
Fever	73.81	65.91	66.14	85.71	68.06	68.87
Cough	69.05	77.27	63.78	50	62.5	69.54
Coryza	32.14	40.91	36.22	35.71	36.11	37.09
Retroocular pain	25	31.82	21.26	50	25	28.8
Headache	59.52	61.36	54.33	64.29	48.61	60.26
Sore throat	39.29	36.36	28.35	42.86	27.78	40.4
Chest pain	36.9	47.73	33.07	42.86	40.28	37.75
Abdominal pain	27.38	22.73	17.32	28.57	25	19.21
Myalgia	60.71	63.64	51.18	57.14	54.17	58.28
Nausea	22.62	36.36	22.05	50	19.44	26.49
Vomiting	13.1	11.36	11.81	7.14	11.11	11.26
Diarrhea	45.24	45.45	38.58	42.86	44.44	41.06
Dyspnea	46.43	56.82	42.52	64.29	50	46.36
Weakness	51.19	61.36	46.46	42.86	48.61	54.97
Fatigue	57.14	65.91	55.12	64.29	51.39	62.91
Anosmia	45.24	50	46.46	57.14	48.61	47.68
Ageusia	44.05	54.55	43.31	64.29	48.61	45.7

N = sample size.

## Data Availability

The raw data supporting the conclusions of this article will be made available by the authors without undue reservation.

## Supplementary Materials

The following supporting information can be downloaded at: https://www.mdpi.com/article/10.3390/v15051197/s1, Supplementary Table S1. Minor Allele Frequencies of studied SNP in different ethnic worldwide populations were obtained from the ENSEMBL database [18,19,20] and in Non-Hospitalized and Hospitalized patients subgroups, according to their ethnic classification.
