# Supplementary material for: Association of Polymorphisms of IL-6 Pathway Genes (IL6, IL6R and IL6ST) with COVID-19 Severity in an Amazonian Population
Source: Viruses. 2023 May 19;15(5):1197. doi: 10.3390/v15051197 (PMC10220739; doi:10.3390/v15051197)
Supplement: Supplementary file 1 [file viruses-15-01197-s001.zip › viruses-2344430-supplementary.pdf]

Supplementary Table S1. Minor Allele Frequencies of studied SNP in different ethnic worldwide populations obtained from ENSEMBL database [18-20] and in Non-Hospitalized and Hospitalized patients subgroups, according their ethnic classification.

Population	rs1800795 [C]	rs2228145 [C]	rs7730934 [A]
Europe	0.42	0.36	0.112
Africa	0.018	0.087	0.508
America	0.184	0.536	0.12
NHSP	0.13	0.4	0.2
NHSP White	0.25	0.38	0.16
NHSP Black	0.17	0.37	0.24
NHSP Brown	0.23	0.41	0.16
HSP	0.17	0.42	0.18
HSP White	0.28	0.42	0.15
HSP Black	0.15	0.3	0.25
HSP Brown	0.3	0.43	0.2

There are no statistically significant differences (Fisher exact test) between Whites, Blacks and Browns subgroups of both NHSP and HSP groups. NHSP=non-hospitalized; HSP=hospitalized.
